# Melting and Vaporization of the 1223 Phase in the System (Tl-Pb-Ba-Sr-Ca-Cu-O)

**DOI:** 10.6028/jres.101.066

**Published:** 1996

**Authors:** L. P. Cook, W. Wong-Ng, P. Paranthaman

**Affiliations:** National Institute of Standards and Technology, Gaithersburg, MD 20899-0001; Oak Ridge National Laboratory, Oak Ridge, TN 37831

**Keywords:** melting point, phase equilibria, thallia vapor pressure, Tl-Pb-Ba-Sr-Ca-Cu-O system, 1223 superconductor

## Abstract

The melting and vaporization of the 1223 [(Tl,Pb):(Ba,Sr):Ca:Cu] oxide phase in the system (Tl-Pb-Ba-Sr-Ca-Cu-O) have been investigated using a combination of dynamic methods (differential thermal analysis, thermogravimetry, effusion) and post-quenching characterization techniques (powder x-ray diffraction, scanning electron microscopy, energy dispersive x-ray spectrometry). Vaporization rates, thermal events, and melt compositions were followed as a function of thallia loss from a 1223 stoichiometry. Melting and vaporization equilibria of the 1223 phase are complex, with as many as seven phases participating simultaneously. At a total pressure of 0.1 MPa the 1223 phase was found to melt completely at (980 ± 5) °C in oxygen, at a thallia partial pressure (*p*_Tl2O_) of (4.6 ± 0.5) kPa, where the quoted uncertainties are standard uncertainties, i.e., 1 estimated standard deviation. The melting reaction involves five other solids and a liquid, nominally as follows:

$$\begin{array}{c} 1223\to 1212+{\left(\text{Ca},\text{Sr}\right)}_{2}{\text{CuO}}_{3}+\left(\text{Sr},\text{Ca}\right){\text{CuO}}_{2}\\ +{\text{BaPbO}}_{3}+\left(\text{Ca},\text{Sr}\right)O+\text{Liquid} \end{array}$$

Stoichiometries of the participating phases have been determined from microchemical analysis, and substantial elemental substitution on the 1212 and 1223 crystallographic sites is indicated. The 1223 phase occurs in equilibrium with liquids from its melting point down to at least 935 °C. The composition of the lowest melting liquid detected for the bulk compositions of this study has been measured using microchemical analysis. Applications to the processing of superconducting wires and tapes are discussed.

## 1. Introduction

### 1.1 Background

The TlBa_2_Ca_2_Cu_3_O_x_ (1223) phase was the first superconducting composition to be reported among its analogs, with a superconducting transition temperature, *T*_c_, of ~ 112 K [1,2]. This compound is well known as one of the Ruddelsden-Popper series of homologous compounds, TlBa_2_Ca*_n_*_−1_Cu*_n_*O_2_*_n_*_+3_ where *n* = number of Cu-O layers [3], in which an alternating structural arrangement of rocksalt and perovskite layers is found. TlBa_2_Ca_2_Cu_3_O*_x_* is tetragonal with space group I4/mmm, *a* = 3.847 Å and *c* = 15.89 Å [4]. The TlBa_2_Ca_2_Cu_3_O*_x_* phase has also been studied in terms of phase equilibria [5,6], bulk processing and properties [7], and thin-film processing and characterization [8]. Other high temperature superconductor members in this series include the 2212, 2223, 2234, and 1234 phases. All are potentially of commercial interest, as their *T*_c_ values (highest ≈ 125 K) are significantly higher than those of Ba_2_YCu_3_O_6+_*_x_* (≈ 90 K) and the Bi-Sr-Ca-Cu-O compounds (highest ≈ 110 K).

Many substitutional variations of the 1223 Tl-Ba-Ca-Cu oxide phase have been studied. Matsuda et al. [9] prepared TlSr_2_Ca_2_Cu_3_O_9_ and found it to have a critical temperature of 100 K. Single phase TlSr_2_Ca_2_Cu_3_O_9_ is known to be difficult to prepare, however the liquid phase synthesis method reported by Morgan et al. [10], has led to some success. The properties and stability of the 1223 family of phases can be substantially improved by partial substitution of Pb and/or Bi for Tl in the rocksalt layer [11–19]. Examples of these compositions are Tl_0.5_Pb_0.5_Sr_2_Ca_2_Cu_3_O_8.2_ [16] and Tl_0.64_Pb_0.2_Bi_0.16_Sr_2_-Ca_2_Cu_3_O*_x_* [12]. The structure of (Tl,Pb)Sr_2_Ca_2_Cu_3_O_9_ was investigated by using neutron powder diffraction and electron microscopy [20]. The strontium and lead-containing 1223 compounds are isostructural (I4/mmm) with TlBa_2_Ca_2_Cu_3_O*_x_*, and all have three CuO_2_-sheets separated by (Tl,Pb)O-and Sr_2_O_2_ layers. For the Sr-containing end member, it was reported that substitutions of Ba for Sr in the perovskite layer created pinning centers and thus improved the superconducting properties [21–23]. Subsequently, various processing investigations [24] and phase evolution studies of the (Tl,Pb)(Sr,Ba)Ca_2_Cu_3_O*_y_* phases [22,25] were conducted. A melt-assisted processing method was found by Doi et al. [22] to be an efficient way to introduce pinning centers into Tl-based superconductors.

Relative to many other high *T*_c_ materials, including the double rocksalt layer thallium compounds, the 1223 phases have special advantages for wire and tape applications due to their high irreversibility line. It has been proposed that this is related to the thinness of the rock-salt layer, with single thallium layer compounds having better performance under applied field due to the increased coupling of the perovskite layers. Superconductor tapes of silver-sheathed (Tl,Pb)(Sr,Ba)_2_Ca_2_Cu_3_O*_y_* have been investigated by Ren et al. [26, 27], and Salazar et al. [28]. The processing of 1223 into wire and tape has been achieved by several alternative methods, including electrophoresis [29], powder in tube [28], and a two-step process including thallination of tape precursors [30]. Silver has been favored as a foundation for the wire or tape, largely because of its promotion in 1223 grain alignment, and the resulting improvement in superconducting properties. Most processing appears to involve a melt phase, either as a transient liquid, or as an equilibrium product. Information on melting equilibria of the 1223 phase is therefore critical for processing. Only a few studies of melting in the Tl-Ba-Ca-Cu-O and related 1223 systems have been investigated, probably due to the high volatility. Blaugher [29] reported a schematic melting diagram based on data available, but included only double thallium layer compounds. Li et al. [31] reported a peritectic melting of unsubstituted 1223 at 905 °C in oxygen. Melting products included CaO and BaCuO_2_.

In addition to melting, a major factor is the control, or monitoring, of thallia pressures during processing. Many of the “1223” systems currently of interest contain six or more components, creating a need for accurate data relating the thallia pressures to multicomponent bulk compositions. Holstein [32] measured thallia pressures over pure Tl_2_O_3_. This serves as a baseline with which to compare pressures in various Tl-containing systems, and has also provided a method for control of Tl pressures, via the “two-zone” method used by Aselage et al. [5] and others. Aselage et al. [5] reported a stability region for the 1223 in terms of the variables *P*_Tl2O_, *P*_O2_ and temperature. A complementary approach would be to measure equilibrium pressures over 1223 as a function of temperature. Wahlbeck et al. [33] measured thallia pressures over the 2223 phase using mass spectrometry. However, thallium pressures over “1223” stoichiometries apparently have not been measured by any of these or any other methods.

One of the central problems of 1223 research concerns stoichiometry. In earlier work [34] we presented preliminary data showing nonstoichiometry of the Tl-Ba-Ca-Cu-O 2212 phase. This is undoubtedly true for 1223 as well; for example, Holstein et al. [8] have presented evidence for a range of disordered phases between 2223 and 1223. It is reasonable to suggest that many of the difficulties encountered in synthesis of single phase 1223 could be explained by compositional shifts associated with nonstoichiometry. On purely crystal chemical grounds such nonstoichiometry would be likely because of the similar ionic radii of Tl^+1^ and Ba^+2^ and of Tl^+3^ and Ca^+2^. Any of these variations would necessarily be linked to oxygen pressure. In principle, Pb^+2^ could substitute on any of three sites, in the rocksalt layer, as well as for Ca and Sr, leading to further stoichiometric variations.

### 1.2 Approach

Of the many 1223 formulations, the (Ba,Pb)-substituted version has shown the greatest potential for further development. Consequently, we have selected the 1223 phase of the Tl-Pb-Ba-Sr-Ca-Cu-O system as the focus for our study. With six cations, this formulation is more tractable than the Bi and Ag-containing systems with eight or more components, and yet it forms a more realistic basis for much of the current processing activity than would the simpler four component analogs. This relatively direct approach of studying a 1223 composition of practical interest does not eliminate the need for data on more basic subsystems, but will serve to highlight particular directions for such activities. The present effort also makes a direct contribution to the processing database for the selected system.

In addition to the large number of phases and the complexity of potential equilibria among them, the study of a multicomponent system such as Tl-Pb-Ba-Sr-Ca-Cu-O presents another difficulty: the lack of easily visualizable general representations, due to the extended array of compositional components which must be treated. This can be partially overcome by using phase equilibrium theory as a guide to regions of low thermodynamic variance, i.e., invariant, univariant and divariant equilibria, which can by definition be conveniently represented in graphical form. Although the compositional parameters may still be intractable, the plotting of univariant reaction lines and invariant reaction points on a p, t grid provides an unambiguous and highly useful representation of the data which constitute the multi-component “phase diagram.” The compositions of all phases, even though it is difficult to represent their combinations graphically, are nonetheless precisely defined along each univariant line or at each invariant point. The Tl-Pb-Ba-Sr-Ca-Cu-O system is a six component system if the oxygen pressure is treated as an externally controlled variable (e.g., *p*_O2_ fixed at 0.1 MPa). This means that each univariant equilibrium (at constant *p*_total_) will contain six phases and each invariant point will involve seven phases. The invariant melting of 1223 must involve seven phases, six of which are presumably solids. It would be expected that from each invariant point will emanate seven univariant lines, each representing an equilibrium between six of the phases participating in the invariant reaction.

## 2. Experimental Methods

### 2.1 Materials Preparation

Three compositions were prepared using carbonate-free oxides, as described in Ref. [25]. These mole fractions, *n*_B_, are given in Table 1a. The combined standard uncertainty, i. e., estimated standard deviation, of each mole fraction is 0.0001. It was found that the use of carbonate-free oxide starting materials greatly reduced processing time by avoiding the difficult kinetics normally associated with reacting alkaline earth carbonates at these temperatures.

Calcining in oxygen of the three compositions yielded 1223, with additional second phases as indicated in Table 1b. Prior to, during, and after the synthesis, all materials handling was completed in a glovebox. The sintered samples were ground and portions set aside for thermal analysis and other experimentation.

### 2.2 Thermal Analysis

The thermal analysis included differential thermal analysis (DTA), thermogravimetric analysis (TGA), and differential thermogravimetric analysis (DTG). For these experiments, samples of from 50 mg to 300 mg were placed in MgO crucibles. The reference was alumina or platinum powder and the DTA assembly was calibrated against the melting point of gold and the alpha/beta quartz transition. The heating rate was 10 °C/min; slower rates were not used due to the increased thallia loss. During the experiments, oxygen was flowed up around the sample at a rate such that thallia condensation on the cooler parts of the DTA assembly was prevented. DTA and DTG event temperatures were chosen on heating at the intersection of the extrapolated baseline with the extrapolated linear portion of the rising peak. Combined standard uncertainties in the thermal analysis event temperatures are estimated at < ± 6 °C.

### 2.3 Effusion Studies

**Fig. 1:**
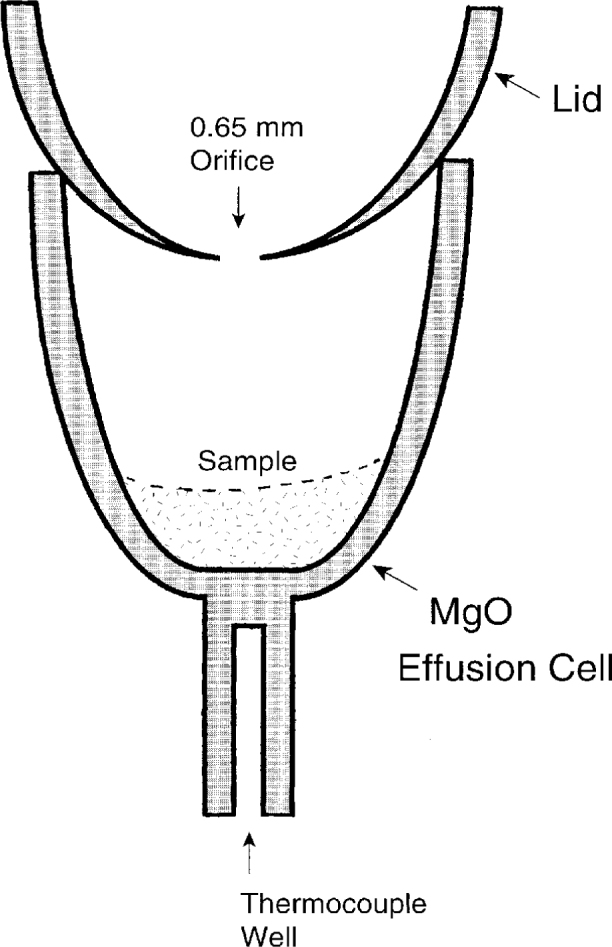
Schematic diagram of effusion cell used in measurement of vaporization rates.

Vaporization rates were obtained thermogravimetrically by a modified effusion method. The effusion cell consisted of a 10 mm diameter MgO DTA cell adapted to accept a tight fitting MgO lid with a 0.65 mm diameter orifice (Fig. 1). In the Knudsen effusion method [35], absolute vapor pressures, *p*, are obtained by measuring mass loss through the orifice according to:

$$P=\frac{1}{A_{o}W^{\prime }}\frac{dw}{dt}\sqrt{\frac{2\pi k_{B}T}{m}}$$ (1)

where *A*_0_ is the area of the circular orifice, d*W*/d*t* is the mass loss per unit time, *W′* is the transmission coefficient of the cell, *m* is the relative molecular mass, *k*_B_ is the Boltzmann constant, and *T* is the temperature in kelvins. This method works well in the molecular flow regime, at pressures < 1 Pa. Wahlbeck et al. [36] have developed a theory which allows the extension of this approach into the transition region between molecular and hydrodynamic flow, over the range 1 Pa to 600 Pa. The upper limit of this range falls within the range of thallia pressures prevalent in many superconductor processing situations. Thus it is of potential application to the equilibrium studies of these materials. Our approach has been to use a calibrant in the effusion cell (in this case pure Tl_2_O_3_) to limit the uncertainties, and still provide an accurate estimate of 1223 thallia pressures. By comparing the ratio of the unknown to the calibrant, most of the parameters in Eq. (1) cancel out, leaving

$$p/p^{*}=\left(dw/dt\right)/{\left(dw/dt\right)}^{*}$$ (2)

where p^*^ and (d*W*/d*t*)^*^ refer to the partial pressure and rate of weight loss, respectively, of the calibrant. The principal uncertainty in this approach is whether *W′* = W′^*^. We believe that the assumption that *W′* = W′^*^ is justified because calibrant and unknown have pressures relatively close to each other, and also because we have used an orifice with “nearly ideal” geometry in these experiments (Fig. 1). The factors determining the transmission coefficient are governed largely by the geometry of the orifice, especially the length, in situations where cylindrical orifices are used. As shown in Fig. 1, the orifice used by us had negligible length, due to its knife-edge geometry. Therefore the transmission factor was unlikely to change significantly for unknown vs calibrant, especially at the higher pressures encountered in these experiments.

**Fig. 2:**
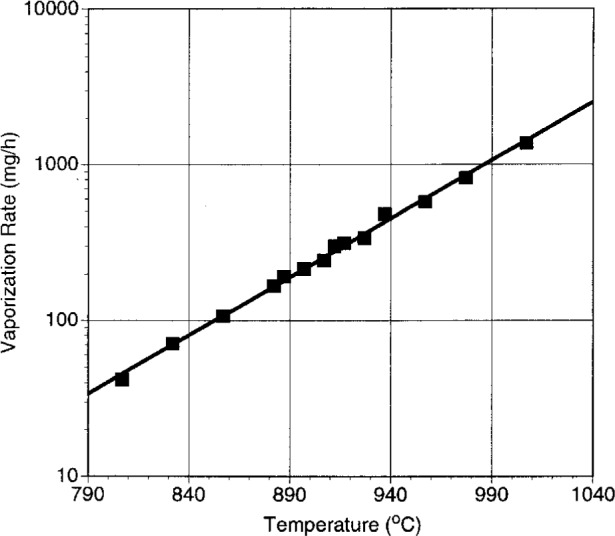
Calibration data for effusion cell, using pure Tl_2_O_3_.

The calibration was achieved by using the same orifice with pure thallia as was used with the samples to be measured. Calibration data are shown in Fig. 2 as a plot of vaporization rate vs temperature for pure Tl_2_O_3_. Below 800 °C (not shown) the data indicated a sharp change in slope. This can be directly related to the data of Holstein [32], who noted that the Tl_2_O_3_ decomposition reaction changes at this temperature. The break in slope is thought to be associated with the onset of melting of Tl_2_O_3_. We observed definite indications of melting in calibration experiments above this temperature, including the presence of meniscii.

The data in Fig. 2 can be correlated with the data of Holstein [32] to obtain a calibration factor as follows. The vaporization reaction is

$${\text{Tl}}_{2}O_{3}\to {\text{Tl}}_{2}{\text{O}+\text{O}}_{2}$$ (3)

with an equilibrium constant given by

$$k=\left(p_{\text{Tl}2O}\right)\left(p_{O2}\right).$$ (4)

Data for Eq. (3) are represented by the expression,

$$\log_{10}k=17.65-20.62\times 10^{3}\;K/T.$$ (5)

The data in Fig. 2 can be fit by the expression

$$f\left(t\right)=\left[\left(4.533434\times 10^{-5}\right)\left(e^{\left(0.0171329\right)t/{}^{\circ}C}\right)\right]\left(\text{mg}/h\right),$$ (6)

where f(*t*) is the vaporization rate at the Celsius temperature *t*. By applying Eqs. (5) and (6) at 794 °C (the upper and lower limits of applicability, respectively) a calibration factor of 58.3733 Pa · mg^−1^ · h is obtained, with a combined standard uncertainty of ± 0.5 Pa · mg^−1^ · h. This factor has been used to convert weight loss data obtained with our thermogravimetric effusion method into thallia pressures.

### 2.4 Microstructural and Microchemical Analysis

Polished sections of experimental samples, including melt-infiltrated MgO wicks, were examined by scanning electron microscopy (SEM). Use of a high gain, low noise backscattered electron detector was found to be essential in differentiating phase regions from one another in the crystalline residual matrix. Compositions of phases were analyzed by energy dispersive x-ray spectrometry (EDS) according to methods described previously [37], at 20 kV or 30 kV and 1 nA beam current. The analytical standards were Tl_2_O_3_, the 4334 Bi:Sr:Ca:Cu oxide superconductor, lead zirconate titanate with 53:47 Ti:Zr, and Ba_2_YCu_3_O_6+x_. Uncertainties are estimated as follows: for mole fractions > 10 %, < ± 5 % relative expanded uncertainty; for mole fractions < 10 %, < ± 10 % relative expanded uncertainty.

### 2.5 Quenching Studies

**Fig. 3:**
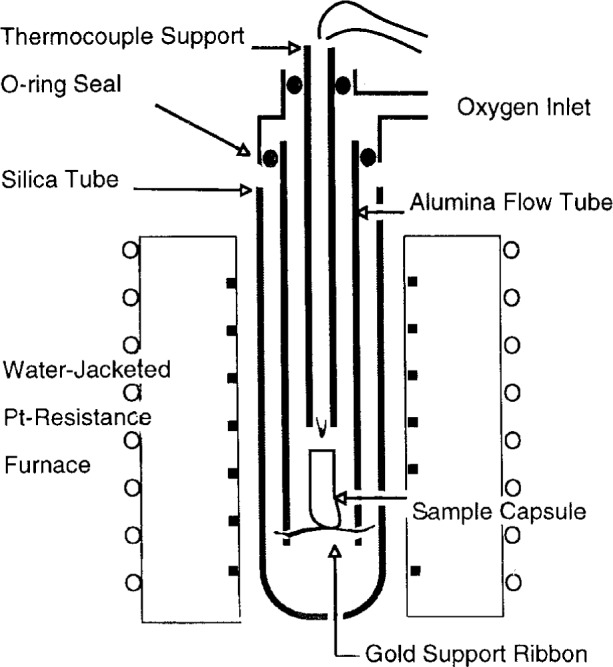
Schematic diagram of improved quench apparatus.

Experiments using the quench method were completed in pure oxygen as described in [38], with an improved apparatus shown in Fig. 3. This apparatus was loaded in a glovebox with a Au sample capsule containing the powdered reactants. A tightly fitting MgO plug was placed in the open end of the capsule to minimize thallia loss. For transport to the furnace, the entire assembly was sealed with o-ring closures, pumped out and backfilled with oxygen. The bottom closure (not shown) was removed, and the assembly was then placed in the hot zone of the furnace with a continuous flow of oxygen out the bottom of the alumina flow tube. At the conclusion of the experiment, the entire assembly was quickly removed from the furnace. Next the top O-ring was loosened, and the thermocouple support was rapidly pushed downward against the capsule to break the gold support ribbon underneath, resulting in the forceful ejection of the sample capsule into a copper cold well. The combined standard uncertainty in the temperature of quench runs is estimated at < ± 5 °C.

### 2.6 Powder X-Ray Diffraction

Solid residues at the conclusion of the experiments were analyzed by x-ray powder diffraction (XRD). All data were collected at room temperature on a Phillips diffractometer^1^ equipped with a theta-compensation slit so as to maintain constant area of x-ray radiation. The data were measured using filtered copper Kα radiation (1.540 598 Å; Deslattes and Henins [39]). Patterns were measured in step-scan mode with a step width of 0.03°. Data collection, peak search (2nd derivative algorithm), and calibration were performed by using the Siemens Diffrac 5000 software suite. Peak indexings and least-squares refinements were obtained by using the NBSLSQ program which was originally written by Appleman and Evans [40].

## 3. Results

### 3.1 Composition 1A (Tl_0.6_Pb_0.4_Sr_1.7_Ba_0.3_Ca_2_Cu_3_O*_x_*)

#### 3.1.1 Sequential Thermal Analysis

**Fig. 4:**
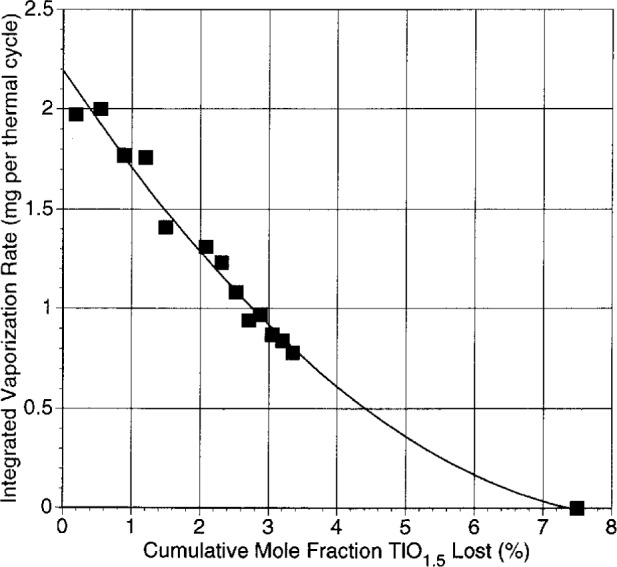
Integrated vaporization data for composition 1A as a function of thallia loss.

**Fig. 5:**
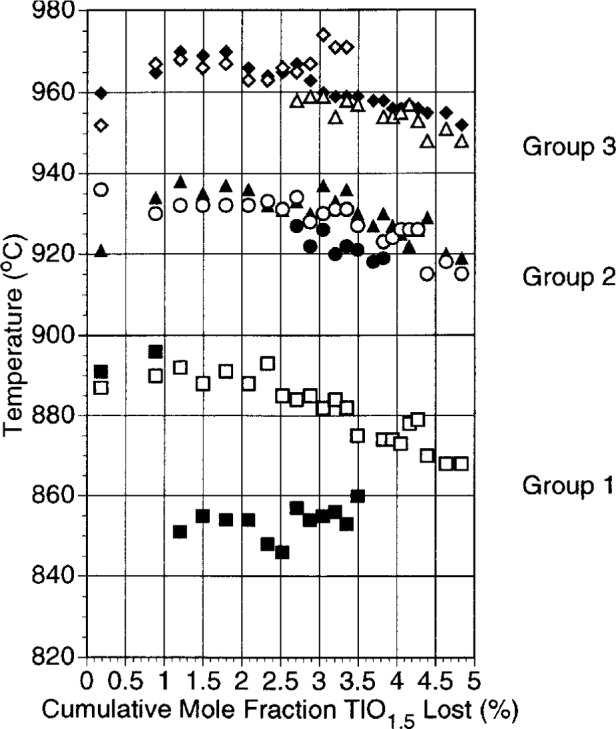
Thermal data for composition 1A as a function of thallia loss. Closed symbols = DTA, open symbols = DTG.

**Fig. 6:**
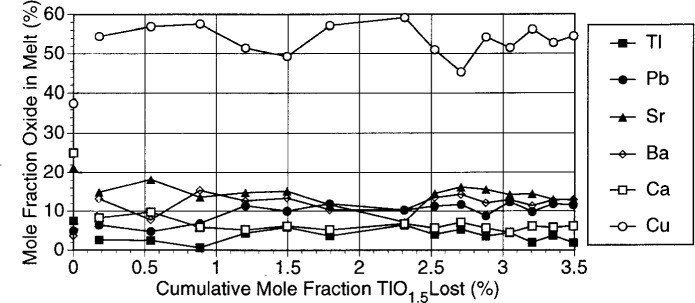
Melt compositions for composition 1A as a function of thallia loss. Bulk composition of starting material for each oxide is at left along vertical axis.

Composition 1A was used for initial experiments to determine the general melting and vaporization behavior of the Tl-Pb-Ba-Sr-Ca-Cu-O system at a nominal 1223 composition. Sequential thermal analysis experiments were completed on composition 1A as follows. A small piece of porous magnesia wick for sampling the melt was placed on top of the powdered sample prior to the thermal analysis, which was completed over the range 800 °C to 1000 °C. On reaching 1000 °C, the power was immediately shut off and the sample was allowed to cool with the furnace. After the sample had reached room temperature, the porous wick with trapped liquid was removed, a fresh wick was added, and the experiment was repeated. This continued, while monitoring thallia loss, until the desired amount of data had been collected, or until other considerations, such as creep of the liquid out of the crucible, required termination of the experiments. In this way a continual accumulation of thermal data on melting and vaporization as a function of thallia loss was obtained (Figs. 4–6).

#### 3.1.2 Vaporization

Data on average thallia vaporization rates are shown in Fig. 4. The data represent the integral of the TlO_1.5_ loss over the range 800 °C to 1000 °C; because of the logarithmic temperature dependence of the vapor pressure, most of the loss occurred at the highest temperature part of the range. The experiments were terminated prior to total thallia depletion, due to melt creep, but a curve has been fit to the data and extrapolated to total thallia loss. For purposes of discussion, if the sample were approximated as a homogeneous solution, the shape of the curve would imply a nearly ideal relation between thallia pressure and bulk composition, with a slight negative departure from ideality. Clearly the samples were not homogeneous, as they were only partially melted. Nonetheless, the general variation in thallia pressures as a function of thallia content for composition 1A is given by Fig. 4. The data allow a smooth extrapolation to total thallia loss, implying that lead loss was not significant in these experiments. This is the same conclusion reached more definitively from vaporization experiments under similar conditions on other (Tl,Pb)-1223 formulations where thallium loss was followed all the way to completion [41].

The absence of well defined plateaus in the data of Fig. 4 indicates the lack of any strong buffering effects due to the presence of multiphase assemblages. During a traverse through multicomponent space such as represented by Fig. 4, multiphase volumes with the number of phases required for univariancy would be intersected (in this case six). The traverse through these volumes would produce plateaus in the vapor pressure curve. Since such plateaus are not evident, it may be that the six phase volumes are very narrow and were not detected at the resolution of Fig. 4. Also the relatively high temperatures at which most of the vaporization of Fig. 4 occurred (near 1000 °C) were in the range where melting increased substantially, possibly eliminating or reducing certain multiphase volumes.

#### 3.1.3 DTA and DTG

Results of DTA and DTG analysis for composition 1A are shown in Fig. 5. Three groups of thermal events can be distinguished: an initial series of events below 900 °C, a second grouping at 915 °C to 940 °C, and a third grouping at 940 °C to 975 °C. In Fig. 5 the DTA and the DTG events correlate reasonably well for the second and third groupings. For the lower temperature events, DTA appears to indicate a different event, at a lower temperature, than DTG. However, events in the lower temperature grouping were very weak and barely detectable. The low temperature DTG event is believed to be associated with the formation of a nonequilibrium eutectic by fractional crystallization during the rapid cooling caused by power shut off at the conclusion of each heating cycle. Upon reheating, this metastable mixture was able to recrystallize to the equilibrium phases, causing the small weight change registered by the DTG. This effect was also observed in composition 1B. The low temperature DTA event is probably associated with a reversible solid state phase change.

Thermal events in the upper two groupings were much stronger. In these two groupings, the correlation between DTA and DTG is quite good. It has been our experience that in the superconducting cuprate systems, melting is always associated with significant weight loss (oxygen) and that therefore the DTG signal can be a fairly sensitive indicator of melting. On this basis we assigned the second series of events to be the onset of melting for the 1223 stoichioimetry of composition 1A in the Tl-Pb-Ba-Sr-Ca-Cu-O system. This was confirmed by independent experiments in which the first penetration of wick by melt with increasing temperature was noted. The third grouping is also a melting reaction, since it is associated with an increased volume of liquid. The thermal data of Fig. 5 indicate substantial complexity, with several closely spaced events in each of the upper two groupings. This complexity appears to have increased somewhat beyond the halfway point in the thallium loss, indicating that additional phase volumes may have been intersected in this region. From Fig. 5, it appears that the general melting behaviour of this particular 1223 stoichiometry, as reflected by the temperatures of the thermal events, did not change dramatically as a function of the thallia loss over the range studied. However, as described below, the assemblage of solids involved in the melting reactions may have changed significantly as thallium was lost.

#### 3.1.4 Melt Compositions and Residual Phases

Data on melt compositions as a function of thallium loss are plotted in Fig. 6. The undulations in the plots of Fig. 6 are greater than the relative standard uncertainties expected in the analyses (generally no more than 5 % to 10 %, as discussed earlier), and appear to be nonrandom. Although more data would be required for specific explanations of the variations in Fig. 6, melt concentrations would be expected to undergo both smooth and abrupt changes, as the boundaries between five-phase and six-phase regions were traversed during thallia vaporization. Moreover, along some parts of the traverse, the thallia content could actually increase in the melt, even as it decreased in the bulk composition. This effect is evident in certain segments of Fig. 6.

In Fig. 6, Cu was enriched from a mole fraction of 37.5 % in the starting stoichiometry to from a mole fraction of 50 % to a mole fraction of 60 % in the liquids. The barium content of the melts was significantly enriched relative to the starting stoichiometry, and generally was in the range of from a mole fraction of 10 % to a mole fraction of 20 % relative to the starting value of a mole fraction of 3.75 %. Pb also increased in the melt phase, from a mole fraction of 5 % in the starting composition to a mole fraction of more than 10 %.

The most strongly depleted oxide was CaO, which although initially present at a mole fraction of 25 %, decreased to from a mole fraction of 5 % to a mole fraction of 10 % in the melts. Thallium was also generally depleted and decreased from the 7.5 mol % present in the starting stoichiometry to nearly zero in the longer term experiments. Although strontium was depleted in all of the melts, this depletion was not as great, from a mole fraction of 21.25 % in the starting material to from a mole fraction of ~ 15 % to a mole fraction of 20 % in the melts.

X-ray powder diffraction analysis of the final product from the vaporization experiments of Figs. 4–6 showed alkaline earth cuprates and plumbates. Additionally, since the experiments were terminated while ~ 50 % of the thallia remained, small amounts of 1223 and 1212 phases were present. With the exception of thallium solid solution in plumbates, there are no known solid phases in the parts of the 1223 system treated in these experiments which contain less thallium than 1223 and 1212. Therefore it would be expected that these phases would coexist with melt up to the final stages of vaporization of the thallium. The detailed behavior of 1223 and 1212 during vaporization must be dependent in part on the degree to which the Pb substitution for thallium in the structures is thermodynamically ideal. In the completely ideal case, thallium would be lost continuously from 1223 and 1212 as well as from the coexisting melt. In practice, this cannot be realized due to the instability of the pure lead end members of Pb-substituted 1223 and 1212. Thus, at some point in the final stages of Tl depletion, 1223 and 1212 must become unstable and the residual thallium is accommodated by the melt phase and by solid solution in plumbates.

In general, the melt compositions in Fig. 6 do not show dramatic change as a function of thallium loss from the bulk compositions. This may be attributed to the crossing of relatively few, or very closely spaced multiphase volumes during thallia depletion. It is noteworthy that situations could arise wherein the crossing from one multiphase stability region into another results in complete crystallization of the melt phase, followed by formation of a new melt phase compositionally unrelated to the first. The fact that no such discontinuities are seen in Fig. 6 suggests that this has not happened; this effect would be expected at temperatures just above the onset of melting where the melt phase regions are separated from one another by nonmelted multiphase assemblages.

### 3.2 Composition 1B (Composition 1A + 10 % Minimum Melt)

The melt data in Fig. 6 represent averages of melt compositions produced over the melting ranges indicated by the thermal events of Fig. 5. They can be used to estimate the compositional identity of the multiphase volume participating in the generation of liquids in equilibrium with 1223 in the thallium deficient part of the phase diagram. For example, if a composition were made up based on melt data in which a mole fraction of 10 % of initial melt phase from Fig. 6 is added to the Tl_0.6_Pb_0.4_Sr_1.7_Ba_0.3_Ca_2_Cu_3_O*_x_* stoichiometry, then the resulting composition can be studied isothermally to determine solid/liquid equilibria at the initiation of melting in the thallia deficient region. This is the region of the phase diagram encountered during processing, if slight depletion of thallia from 1223 stoichiometries occurs. The 1223/melt composition corresponding to composition 1B (see Table 1b) was made up accordingly.

Table 1b shows results of annealing composition 1B at 900 °C, which produced a mixture of 1212, 1223, CuO, CaO, BaPbO_3_, and (Sr,Ca)_14_Cu_24_O_41_. After melting at 945 °C, the CuO had disappeared and (Ca,Sr)_2_PbO_4_ and (Ca,Sr)_2_CuO_3_ had formed. As a general observation, the appearance of the (Ca,Sr) plumbate phase was associated with melting of 1223 and related compositions and it usually formed during rapid cooling from the melt. Also, melting generally resulted in the appearance of additional calcium cuprates, along with the disappearance of CuO. Thus, the first appearance of liquid in 1223 stoichiometries may result from a univariant reaction among seven phases, with CuO on the low temperature side and liquid on the high temperature side. This liquid for composition 1B has been sampled about 10 °C above the minimum melting temperature of 935 °C, and the infiltrated wick analyzed quantitatively by EDS. The result is given in Table 2.

The liquid composition of Table 2 was prepared; nearly complete melting was observed, consistent with its existence as a stable phase at 935 °C. It corresponds to the first liquid to appear in contact with 1223 for bulk composition 1B. The liquid appears below 935 °C, and it forms initially at 905 °C, indicating that its initial appearance in contact with 1223 is not a eutectic phenomenon. Upon rapid cooling and reheating, a DTA/DTG event was observed at 880 °C. After annealing at 915 °C, the 880 °C event was no longer observable by DTA or DTG, suggesting that it resulted from the reaction of a eutectic melt trapped by rapid cooling and fractional crystallization. It does not represent equilibrium melting for composition 1B, but rather, only a transient effect. This eutectic melt may not be stable in contact with 1223 for bulk composition 1B, and therefore the non-eutectic melt of Table 2 is the first melt to appear in contact with 1223. The melt of Table 2 probably appears within the six phase volume: CuO + BaPbO_3_ +1212+1223+(Ca,Sr)_2_CuO_3_+(Sr,Ca)_14_Cu_28_O_41_, which apparently gives way to the six phase volume BaPbO_3_ + 1212 + 1223 + (Ca,Sr)_2_CuO_3_ + (Sr,Ca)CuO_2_ + Liquid or CaO + 1212 + 1223 + (Ca,Sr)_2_CuO_3_ + (Sr,Ca)CuO_2_ + Liquid at higher temperatures by a series of invariant melting reactions, as discussed for composition 2 below.

### 3.3 Composition 2 (Nearly Single Phase 1223)

#### 3.3.1 Formation of the 1223 Phase

**Fig. 7:**
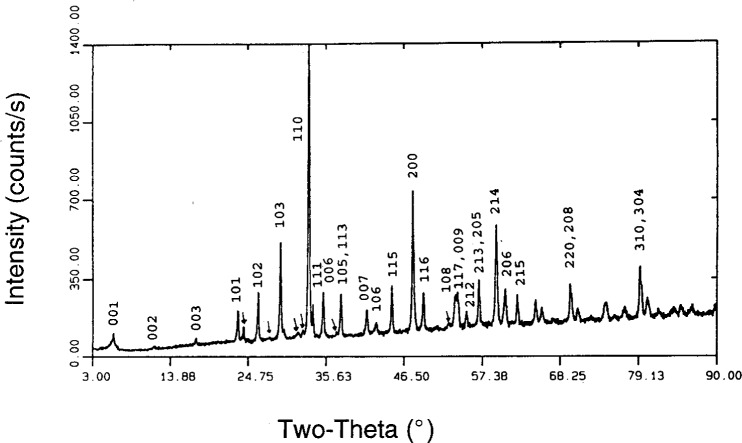
XRD pattern of nearly single phase 1223, Tl_0.72_Pb_0.56_Ba_0.16_Sr_1.91_Ca_1.74_Cu_3_O*_x_*, resulting from calcining of composition 2. The symbol ↓ is used to indicate impurity phases.

**Fig. 8:**
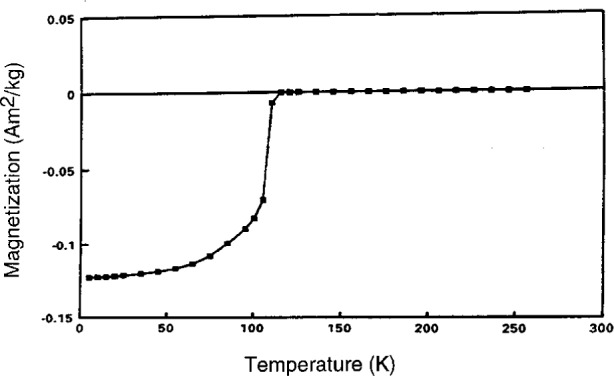
Magnetic susceptibility data for 1223 phase in Fig. 7, indicating a *T*_c_ onset of 115 K.

Composition 1B, while useful for investigation of initial melting, did not contain enough 1223 phase to conduct studies of the upper melting point of 1223. Therefore, based on EDS analyses of the 1223 phase which had crystallized in composition 1B and its derivatives, attempts were made to prepare compositions with a substantially higher percentage of 1223. Several attempts succeeded in preparing only 1212. This suggests that 1212 may have a substantial homogeneity range, which under certain circumstances may actually overlap the 1223 range. This was made apparent during the preparation of composition 2. The first heat treatment at 950 °C for 15 min gave more 1212 than 1223. Subsequent comminutions and reheats increased the percentage of 1223 to the point where the 1212 had almost totally vanished leaving a nearly single phase 1223. Figure 7 shows the x-ray diffraction pattern of this nearly single phase 1223, with the (*hkl*) values of some major peaks labelled. In this sample, only very minor amounts of other phases such as (Sr,Ca)_14_Cu_24_O_41_, were detected, as indicated. The nominal unit cell parameters for this material were found to be *a* = 3.8142 ± 0.0012 Å, *c* = 15.304 ± 0.005 Å, and *V* = 222.6 ± 0.1 Å^3^, with a space group of I4/mmm, where the uncertainties listed are standard uncertainties. The composition of the phase, as estimated from EDS analysis, was Tl_0.72_Pb_0.56_Ba_0.16_Sr_1.91_Ca_1.74_Cu_3_O_x_. If the standard site-occupancy scheme is used, this requires a certain amount of intersite substitution, probably lead for calcium. This preparation had a relatively sharp *T*_c_ onset of 115 K (Fig. 8), confirming the presence of 1223 as the dominant superconductor. The vanishing of 1212 during annealing of composition 2 may have been partly, but not totally, due to thallium vaporization during the reheats, as thallium loss was compensated during preparation of a large batch with little change in result. A small loss of thallium did, however, slightly diminish the quantity of second phase 1212 for composition 2. The dominant factor in the disappearance of initially formed 1212 is thought to have been its metastability relative to 1223.

#### 3.3.2 Melting Point of 1223

The high percentage of 1223 in composition 2 made it feasible to conduct melting point studies. Regardless of impurity phases, the complete melting of 1223 should be an invariant process at constant *P*_O2_ and *P*_total_. This requires that a single, well defined 1223 composition be present at the melting point. The impurity phases would interfere if 1223 melted congruently, but this is definitely not the case, as no melts observed at temperatures up to its disappearance have a composition approaching 1223.

**Fig. 9:**
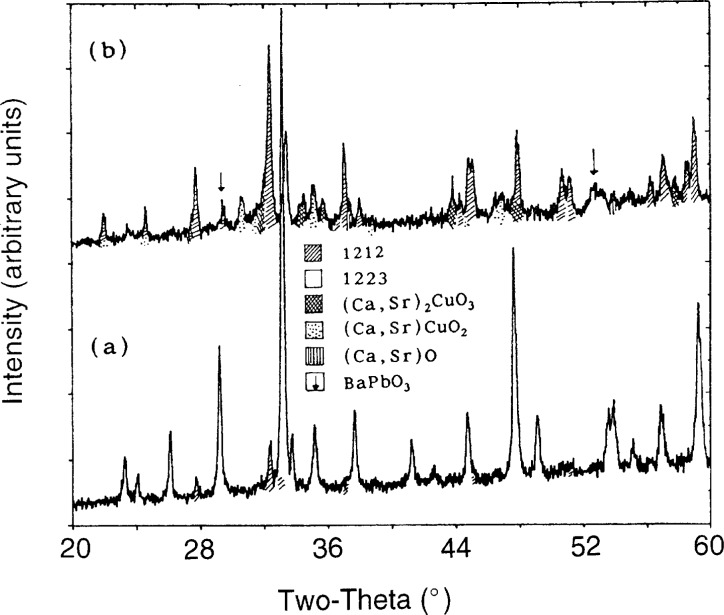
XRD patterns of composition 2, (a) before (975 °C) and (b) after (980 °C) melting of the 1223.

**Fig. 10:**
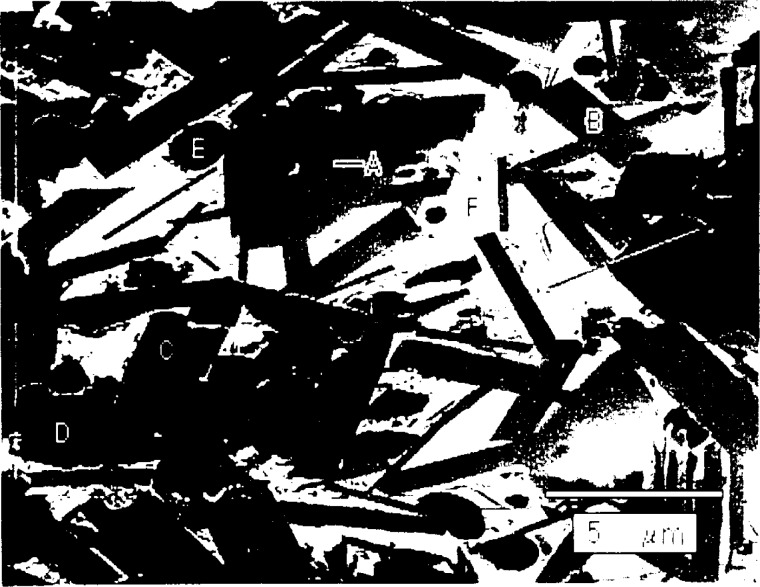
Backscattered electron micrograph, showing microstructure of composition 2 quenched from 980 °C. A = residual 1223; B = 1212; C = (Sr,Ca)_2_CuO_3_; D = (Ca,Sr)CuO_2_; E = (Ca,Sr)O; F = melt.

The melting point of 1223 was investigated in two stages. First, after careful annealing (three times at 950 °C), DTA/DTG was completed on composition 2, indicating an initial melting at 932 °C and another major melting event at 978 °C. Next, quenching studies were completed at five degree intervals on composition 2, and the products analyzed by XRD and SEM/EDS. The XRD data in Fig. 9 indicate that 1223 was present at 975 °C, but not at 980 °C. Examination of the 980 °C experiment by SEM/EDS showed less than 2 % 1223 remaining, mostly as vestigial grains completely surrounded by 1212 (Fig. 10). Clearly the melting point was very close to this temperature, with a value chosen as (980 ± 5) °C. Phases participating in the melting reaction were 1212, CaO, (Sr,Ca)CuO_2_, (Ca,Sr)_2_CuO_3_, BaPbO_3_, and liquid. BaPbO_3_, while not observed in the area of the micrograph, was observed in the x-ray pattern. BaPbO_3_ must participate in the invariant melting, because of its involvement in univariant reactions emanating from the melting point. The postulated melting reaction is:

$$\begin{array}{c} 1223\to 1212+{\left(\text{Ca},\text{Sr}\right)}_{2}{\text{CuO}}_{3}+\left(\text{Sr},\text{Ca}\right){\text{CuO}}_{2}+{\text{BaPbO}}_{3}+\\ \left(\text{Ca},\text{Sr}\right)O+\text{Liquid} \end{array}$$ (7)

**Fig. 11:**
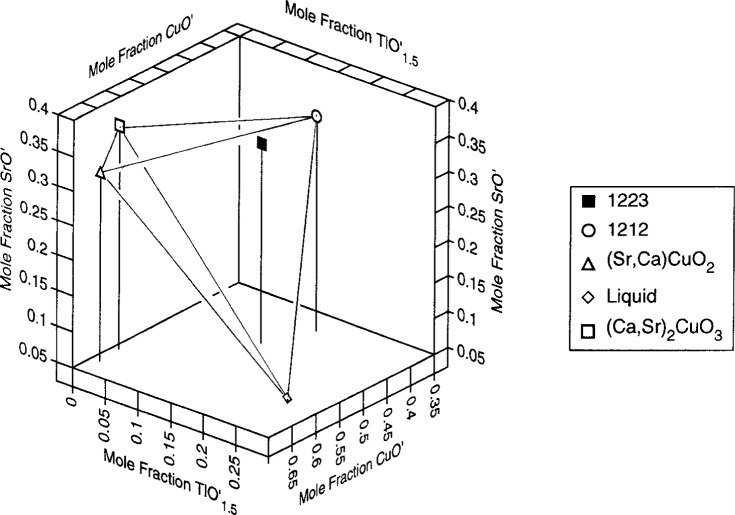
Phase diagram of 1223 melting at 980 °C. Compositions of coexisting phases have been projected through CaO and BaPbO_3_ and mapped onto CuO′-TlO_1.5_′-SrO′ compositional coordinates, where mole fraction CuO′ + mole fraction TlO_1.5_′ + mole fraction SrO′ + mole fraction PbO′ = 1.

Compositions of phases participating in Eq. (7) are given in Table 3a and plotted in the projection of Fig. 11. The 1223 phase plots inside the region bounded by the other phases in Fig. 11, confirming the validity of the incongruent melting reaction. The composition of the 1223 participating in the melting point reaction has a slightly different composition (Table 3b) than the phase in Fig. 7. This is indicative of a solid solution range in the 1223. This range is probably smaller than than that for the 1212, which for this reaction, has an estimated composition of Tl_0.77_Pb_0.56_Sr_1.78_Ba_0.10_Ca_0.84_Cu_2_O*_x_*. The 1212 and 1223 phases are close compositionally to each other, with the result that small changes in bulk composition can have a dramatic effect on the phases present. Both the 1212 and 1223 phases have greater (Tl + Pb) than would correspond to the stoichiometric “1” associated with a fully occupied single rocksalt layer. Some, but not all of the excess (Tl + Pb) could be accommodated by substitution in the Sr and Ca sites. Therefore these phases may be partially disordered, with a certain percentage of double rocksalt layers [8].

The calcium oxide phase in Table 3b contains a mole fraction of nearly 5 % SrO, a mole fraction of more than 5 % TlO_1.5_, and a mole fraction of about 2 % CuO. No significant BaO was found in the calcium oxide. Lead showed extensive substitution for thallium in the 1212 and 1223 superconductors, which have Pb/(Tl + Pb) ratios of 0.42 and 0.41, respectively. The (Ca,Sr)_2_CuO_3_ phase showed very little substitution of Tl, Pb or Ba, unlike the (Sr,Ca)CuO_2_ phase, which accepted a mole fraction of between 1 % to 2 % of each. The melt phase in this reaction contained substantially more thallium and less alkaline earths than the minimum melt of Table 2.

#### 3.3.3 Vapor Pressure

The thallia vapor pressure at the 1223 melting point is an important thermodynamic parameter. It is probably not feasible to measure it directly due to the difficulty in holding the temperature exactly at the required value. However, the task can be simplified by noting that the melting point of the 1223 also represents the intersection of seven univariant lines. Hence, if one of these curves can be measured well enough to extrapolate to 980 °C, the thallia vapor pressure at the melting point can be estimated.

Composition 2 was found to be well suited to this application. A slight loss in thallium puts the composition into the six phase volume: CaO + 1223 + 1212 + (Sr,Ca)CuO_2_ + (Ca,Sr)_2_CuO_3_ + liquid, as shown by quench experiments. This provides a stable univariant combination which can be measured thermogravimetrically at a series of temperatures. To obtain reproducible results, it was found that the sample must be very well homogenized and equilibrated prior to the measurements. Also it was necessary to limit data collection times to less than 0.5 h so that thallia loss did not exceed the limits of the six phase volume. During this time the constancy of the vapor pressure was verified by the linearity of the weight loss curve.

**Fig. 12:**
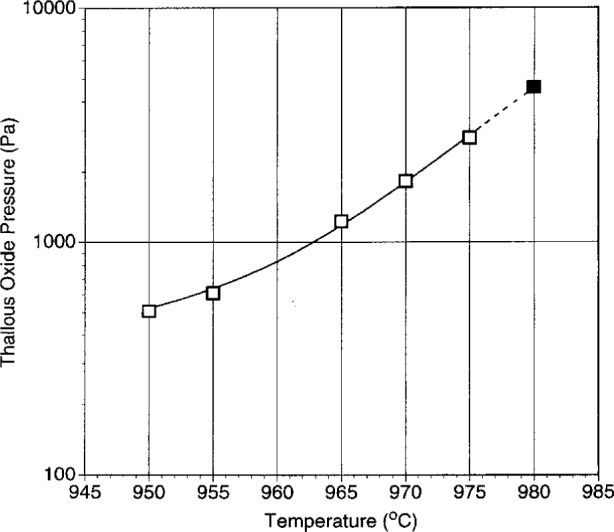
Vaporization data for the 1223 + 1212 + (Ca,Sr)_2_CuO_3_ + (Sr,Ca)CuO_2_ + CaO + melt assemblage as a function of temperature. Dashed line shows extrapolation to the melting point of 1223 at 980 °C.

Vaporization data are shown in Fig. 12, where they have been converted to vapor pressure data by the methods already described. The extrapolated value of the curve at 980 °C is (4.6 ± 0.5) kPa. This represents our best estimate of the thallia pressure (*p*_Tl2O_) at the 1223 melting point.

## 4. Discussion

### 4.1 The 1223 Phase Diagram

The data in Fig. 12 represent an initial contribution to the determination of the 1223 phase diagram. As noted above, the phase diagram is best represented as a *p*_Tl2O_—*t* grid showing the relevant invariant points and univariant lines. The 1223 melting point is perhaps the single most important point on the phase diagram. To reiterate, this point is also the origin (or termination) of many of the relevant univariant lines which define the 1223 stability field. In Fig. 12, we show one of these lines, corresponding to the six phase equilibrium CaO + 1223 + 1212 + (Sr,Ca)CuO_2_ + (Ca,Sr)_2_CuO_3_ + liquid. In principle, there should be six other univariant lines emanating from the melting point. One of these will not include 1223 and will extend to higher temperature, and therefore is not of interest, unless more extensive melting is sought. Based on Fig. 5 and other observations the 1223 + liquid field extends from the melting point at 980 °C down to at least 935 °C. In the lower part of this range, the phase diagram will be complicated by the presence of additional solids and invariant points. It is anticipated that further experimentation will enable extension of the data in Fig. 12, and ultimately the completion of the 1223 phase diagram.

### 4.2 Applications

**Fig. 13:**
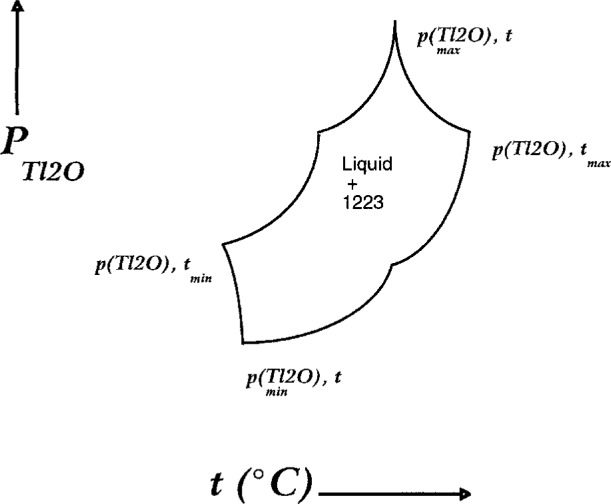
Schematic processing diagram for 1223 indicating hypothetical univariant limits of liquid + 1223 processing region.

Applications can be discussed with reference to Fig. 13, where the aspects of the 1223 phase diagram of most interest to processing are indicated on a hypothetical *p*_Tl2O_ vs temperature plot. All the univariant and invariant equilibria (constant *P*_total_, *P*_O2_) relative to 1223 stability in the Tl-Pb-Ba-Sr-Ca-Cu-O system can be represented on such a plot. The essential features of 1223 equilibria of special importance to processing include the boundaries of the region where melts are in equilibrium with 1223. These are shown schematically in Fig. 13. The maximum extent of the 1223 + liquid field is defined by four (*p*_Tl2O_, *t*) points, labelled as (*p*,*t*_min_), (*p*_max_,*t*), (*p*,*t*_max_), and (*p*_min_,*t*). Within this field 1223 and liquid coexist with other solids, whose number depends on the thermodynamic variance of the equilibrium involved. The points (*p*_max_,*t*) and (*p*_min_,*t*) define the upper and lower vapor pressure limits of the melt processing field. At pressures above or below these limits, 1223 will decompose. The points (*p*,*t*_max_) and (*p*,*t*_min_) correspond to the maximum and minimum temperature limits available for 1223 melt processing. These values are especially important for the control of kinetic factors. If further investigation reveals that (*p*_max_,*t*) = (*p*,*t*_max_), and/or (*p*_min_,*t*) = (*p*,*t*_min_), then a simpler outline for the melt processing region would result.

The experimental curve in Fig. 12 corresponds most closely to the upper part of the (*p*_min_,*t*) − (*p*,*t*_max_) boundary of the 1223 + liquid stability field in Fig. 13. This boundary indicates the most readily accessible region for processing: vaporization is minimized, and yet the beneficial aspects of liquid coexistence are maintained. The data in Fig. 12 apparently represent the highest pressures and temperatures reported for 1223 synthesis. Aselage et al. [24] reported single phase 1223 at 920 °C and *p*_Tl2O_ = 0.45 kPa, with *p*_O2_ = 0.08 MPa, yet the presence of liquid, though suggested, was not confirmed. The use of higher temperatures could enhance grain growth and homogeneity during processing, especially in the presence of a melt phase, provided thallium loss can be controlled. For successful 1223 processing in the liquid + 1223 phase region, accurate control of compositions is essential, especially given the close proximity of the 2212 phase field.

## Figures and Tables

**Table 1a:** Bulk compositions investigated (mole fraction, *n*_B_)

Composition No.	CuO	TiO_1.5_	PbO	SrO	CaO	BaO
1A	0.3750	0.0750	0.0500	0.2125	0.2500	0.0375
1B	0.3920	0.0702	0.0514	0.2062	0.2333	0.0470
2	0.3038	0.0868	0.0608	0.2268	0.2191	0.0227

**Table 1b:** Results of x-ray powder diffraction analysis of the starting materials in Table 1a after annealing

Composition No.	Annealing conditions	Phases present
1A	900 °C, Oxygen	1223, 1212, (Sr,Ca)_14_Cu_24_O_41_ Plus other minor phases
1B	900 °C, Oxygen	1223, 1212, (Sr,Ca)_14_Cu_24_O_41_, BaPbO_3_, CuO, CaO
2	950 °C, Oxygen	1223 (major), 1212 (minor), (Sr,Ca)_14_Cu_24_O_41_

**Table 2:** Minimum melting liquid produced from composition 1B at 946 °C (mole fraction, *n*_B_)

CuO	TiO_1.5_	PbO	SrO	CaO	BaO
0.5218	0.0885	0.1415	0.1093	0.0397	0.0991

**Table 3a:** Compositions of phases at melting of 1223 (mole fraction, *n*_B_)

Nominal Formula	CuO	TiO_1.5_	PbO	SrO	CaO	BaO
1212	0.3304	0.1278	0.0932	0.2935	0.1388	0.0165
(Sr,Ca)CuO_2_	0.5186	0.0107	0.0193	0.2515	0.1843	0.0156
(Ca,Sr)O	0.0223	0.0541	0.0010	0.0480	0.8735	0.0011
Melt	0.5178	0.2212	0.1418	0.0487	0.0058	0.0645
(Ca,Sr)_2_CuO_3_	0.3509	0.0052	0.0050	0.2095	0.4274	0.00
1223	0.3566	0.0940	0.0652	0.2504	0.2175	0.0163

**Table 3b:** Phase Stoichiometries, recalculated from Table 3a

Nominal formula	Calculated structural formula
1212	Tl_0.77_Pb_0.56_Sr_1.78_Ba_0.10_Ca_0.84_Cu_2_O*_x_*
(Sr,Ca)CuO_2_	(Sr_0.49_Ca_0.36_Tl_0.02_Pb_0.04_Ba_0.03_)CuO*_x_*
(Ca,Sr)O	(Ca_0.88_Sr_0.05_Tl_0.05_Cu_0.02_)O
(Ca,Sr)_2_CuO_3_	(Ca_1.22_Sr_0.60_Tl_0.01_Pb_0.01_)CuO*_x_*
1223	Tl_0.79_Pb_0.55_Sr_2.11_Ba_0.14_Ca_1.83_Cu_3_O*_x_*

## Footnotes

1 Certain commercial equipment, instruments, or materials are identified in this paper to foster understanding. Such identification does not imply recommendation or endorsement by the National Institute of Standards and Technology, nor does it imply that the materials or equipment identified are necessarily the best available for the purpose.
